# RNA Encapsidation and Packaging in the Phleboviruses

**DOI:** 10.3390/v8070194

**Published:** 2016-07-15

**Authors:** Katherine E. Hornak, Jean-Marc Lanchy, J. Stephen Lodmell

**Affiliations:** 1 Division of Biological Sciences, University of Montana, Missoula, MT 59812, USA; katherine.hornak@umontana.edu (K.E.H.); jean-marc.lanchy@umontana.edu (J.-M.L.); 2 Center for Biomolecular Structure and Dynamics, University of Montana, Missoula, MT 59812, USA

**Keywords:** bunyavirus, phlebovirus, RNA packaging

## Abstract

The *Bunyaviridae* represents the largest family of segmented RNA viruses, which infect a staggering diversity of plants, animals, and insects. Within the family *Bunyaviridae*, the *Phlebovirus* genus includes several important human and animal pathogens, including Rift Valley fever virus (RVFV), severe fever with thrombocytopenia syndrome virus (SFTSV), Uukuniemi virus (UUKV), and the sandfly fever viruses. The phleboviruses have small tripartite RNA genomes that encode a repertoire of 5–7 proteins. These few proteins accomplish the daunting task of recognizing and specifically packaging a tri-segment complement of viral genomic RNA in the midst of an abundance of host components. The critical nucleation events that eventually lead to virion production begin early on in the host cytoplasm as the first strands of nascent viral RNA (vRNA) are synthesized. The interaction between the vRNA and the viral nucleocapsid (N) protein effectively protects and masks the RNA from the host, and also forms the ribonucleoprotein (RNP) architecture that mediates downstream interactions and drives virion formation. Although the mechanism by which all three genomic counterparts are selectively co-packaged is not completely understood, we are beginning to understand the hierarchy of interactions that begins with N-RNA packaging and culminates in RNP packaging into new virus particles. In this review we focus on recent progress that highlights the molecular basis of RNA genome packaging in the phleboviruses.

## 1. Introduction

The *Phlebovirus* genus represents one of five *Bunyavirus* genera (*Orthobunyavirus*, *Hantavirus*, *Nairovirus*, *Tospovirus*, and *Phlebovirus*). The International Committee on Taxonomy of Viruses cites nine species within the *Phlebovirus* genus, to which at least 70 distinct serotypes belong [1]. The genus can be further divided into two subgroups: Uukuniemi-like and sandfly fever-like viruses. Additionally, there are more than 30 potential *Phlebovirus* genus members awaiting a species designation [1]. Excluding the rodent-borne hantaviruses, most bunyaviruses are carried by arthropod vectors, including mosquitos, ticks, sandflies and thrips, and can infect a diversity of animal and plant host species. Phleboviruses can replicate within susceptible vertebrates and in arthropod vectors, but are cytolytic only in their vertebrate hosts.

Phleboviruses have become medically and economically significant around the world within the past century. The Uukuniemi-like viruses are transmitted by ticks, while the sandfly fever viruses are spread by mosquitoes or phlebotomine flies. Outbreaks caused by the sandfly fever viruses, including sandfly fever Sicilian virus (SFSV), sandfly fever Naples virus (SFNV), were documented during the World War II era, during which epidemics were documented amongst the Allied troops in Italy [2]. SFSV and SFNV are currently widespread throughout the Mediterranean basin, with seroprevalence rates approaching 50% in some regions [2]. Infections can produce intense fever, headaches, nausea, vomiting and diarrhea that may last for several days [3]. Toscana virus (TOSV), also vectored by phelobotomines, was isolated in Italy in the 1970s and has since migrated to surrounding countries including Spain, France, Turkey and Greece [4]. In addition to flu-like symptoms similar to those caused by Sicilian and Naples sandfly fever viruses, TOSV is neuroinvasive and can cause aseptic meningitis and meningoencephalitis [5].

The most studied phlebovirus, Rift Valley fever virus (RVFV), was first isolated in Kenya in the 1930’s and has since spread throughout the African continent and more recently into the Arabian Peninsula [6,7]. The virus can be transmitted by more than thirty mosquito vector species [8], including *Aedes aegypti* mosquitos, which can also carry a number of other human pathogens including Zika, Yellow fever, and Dengue viruses. RVFV infection is endemic to parts of sub-Saharan Africa and the Arabian Peninsula, and has caused notable localized epidemics with significant human mortality and morbidity that coincide with heavy rainfall and the prevalence of mosquito vectors. RVFV causes a wide range of clinical manifestations in humans, from general malaise and flu-like illness to blindness, meningoencephalitis, hemorrhagic fever and death [7,8,9,10,11]. Epidemics also produce intensely destructive abortion storms in livestock, with mortality rates approaching 100% in fetuses and neonates and 10%–20% in adults [12]. In contrast, tick-borne phleboviruses have not historically been considered significant public health threats, although this perception is rapidly changing with the classification of emerging phleboviruses, such as Heartland virus, Bhanja virus and severe fever with thrombocytopenia syndrome virus (SFTSV), which have spread more broadly and have caused severe disease outbreaks in humans [13,14,15,16,17].

## 2. Molecular Biology of Phleboviruses

**Figure 1:**
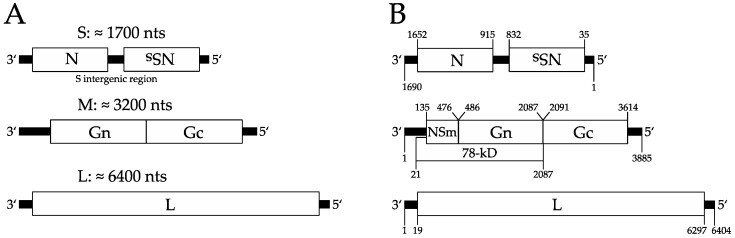
Phlebovirus genome organization. (**A**) The genes encoded in the three strands of a typical phlebovirus genome are represented by open boxes labeled with each gene’s abbreviation and the average size of each strand. The untranslated regions are represented by a thick black bar. The ambisense nature of the S strand is highlighted by the upside down name of the non-structural protein on S segment (NSs); (**B**) The three strands of Rift Valley fever virus (RVFV, strain MP-12) are shown with the nucleotide labels representing each coding region. The L, M, and S strands correspond to the GenBank nucleotide entries, DQ375404, DQ380208, and DQ380154, respectively. In addition to the proteins encoded by all phleboviruses, some, including RVFV, also code for a nonstructural protein called NSm upstream of Gn and Gc, thus explaining the relative increase of the M strand genome length (3885 nts for RVFV vs. a 3200 nts average).

The molecular virology of the bunyaviruses has been actively studied for years, and the major attributes of their replication cycles and interactions with host cells have been described as the result of myriad scientific approaches by many laboratories around the globe [18,19]. The phleboviruses, like all bunyaviruses, have negative or ambisense single-stranded tripartite RNA genomes (see Figure 1). A hallmark of the *Phlebovirus* genus is the use of an ambisense coding strategy in the smallest of the three genome segments, the S segment, whereby the nucleocapsid (N) protein mRNA is transcribed from the viral sense, and the mRNA for the virulence factor, termed non-structural protein on S segment, (NSs), is transcribed from the antisense RNA intermediate [20,21]. The other two genome segments, M and L, encode in negative sense the glycoproteins (Gn and Gc) and the viral RNA (vRNA) dependent RNA polymerase (RdRP or L protein), respectively. In some phleboviruses, an additional nonstructural protein important for viral replication in insect cells, designated NSm, is encoded within the M segment. Notably, the Uukuniemi-like viruses lack NSm altogether [19]. Each segment has a similar organization, with short untranslated regions (UTRs) flanking the longer protein coding central regions. The UTRs have complementary 5′- and 3′-ends with conserved phlebovirus-specific sequences that base pair to form panhandle structures that play a role in vRNA encapsidation and packaging (see Section 6). These circular ribonucleoproteins (RNPs) are visible by electron microscopy [19,22,23]. The UTRs of each segment also contain signals necessary for transcription and replication [24].

Phlebovirus virions exhibit icosahedral symmetry and measure approximately 100 nm in diameter [19]. Virions are composed of Gn and Gc glycoproteins that stud the lipid bilayer envelope, and the three genomic RNPs, which are coated in N protein and complexed with the enzyme L. The virus gains entry into a susceptible host cell via receptor-mediated endocytosis [25]. The host receptor(s) required for entry remain largely unidentified for most phleboviruses, although DC-SIGN has been identified as a receptor in dendritic cells for Uukuniemi virus (UUKV) and RVFV [26]. Clathrin and caveolin dependent endocytic pathways are utilized by phleboviruses [25,27]. Upon low pH acidification of the endosome, the class II fusion glycoprotein Gc mediates fusion of the virion and cell membranes, allowing the virus entrance into the cytoplasm, where primary transcription and replication occur [25,28].

Transcription of vRNA to mRNA requires genomic RNA coated in N protein as a template. Each vRNA segment is accompanied in the virion by the RdRP [29,30]. Priming of mRNA synthesis occurs via cap-snatching, which requires short oligonucleotides with 5′ caps cleaved from cytoplasmic host mRNAs by the endonuclease action of L and perhaps in concert with other viral or host factors [19,31]. It is unclear how the viral polymerase machinery switches from primary transcription, which generates truncated transcripts, to replication mode, which requires that the polymerase synthesize full-length genomic copies destined to end up in progeny virions. However, studies utilizing the prototypical bunyavirus Bunyamwera (BUNV) suggest that N protein plays an important role in that switch [22,32,33,34].

Viral assembly and budding occur at the Golgi apparatus, which is a distinguishing characteristic of bunyaviruses [35]. However, plasma membrane budding has been documented during RVFV infection in rat hepatocytes [36]. The mechanism by which a full complement of large, medium, and small (L, M, S) RNPs are incorporated into nascent virions has yet to be elucidated for any of the phleboviruses or bunyaviruses, and largely remains an open question for other segmented RNA viruses.

**Figure 2:**
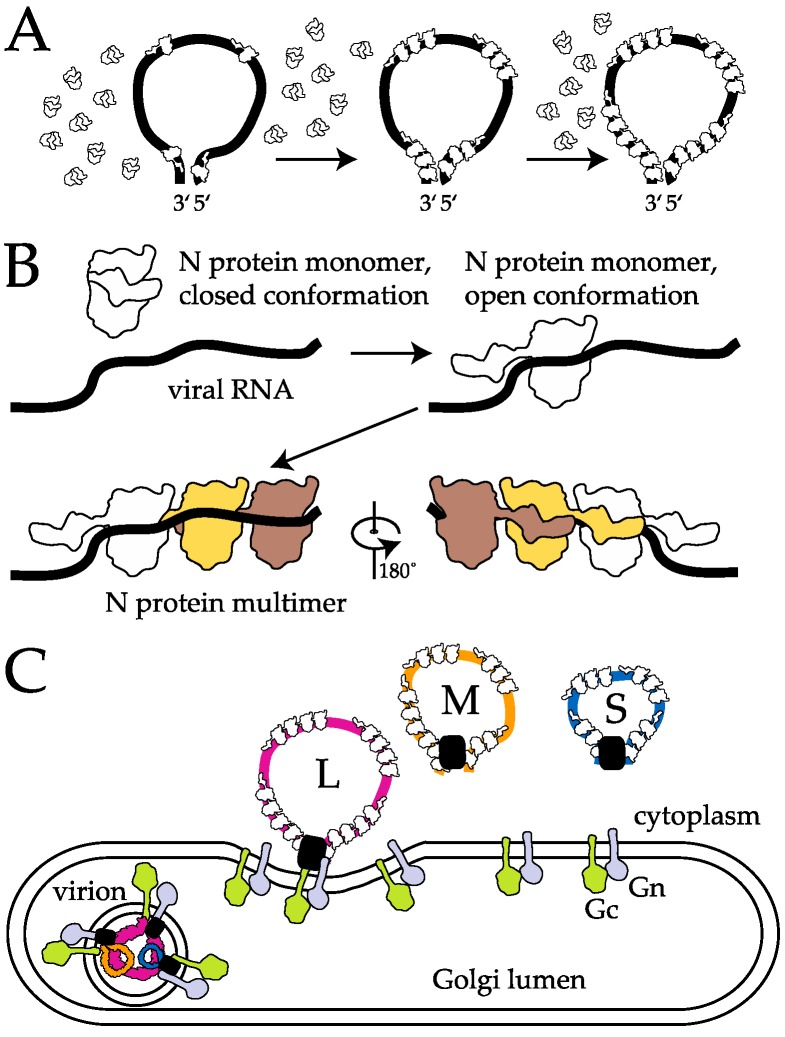
Levels of molecular interactions contributing to RNA packaging during phlebovirus replication discussed in this review. (**A**) Formation of the RNP complex. Genomic RNA segments have complementary 5′ and 3′ ends, contributing to the characteristic panhandle structure. Nucleocapsid (N) proteins, exhibit some sequence and/or structure preference on target RNAs and binds to recognition site(s), nucleating further cooperative N-RNA binding events; (**B**) Model for RNA dependent conformational change of N. The RNA binding cleft of N protein is occluded by the amino-terminal arm until it encounters a cognate RNA binding site. Upon binding, the amino-terminal arm swings open and recruits non-sequence specific binding of further N molecules along the RNA. The back view shows placement of the amino terminal arm of each monomer on the outside hydrophobic cleft of the adjacent N monomer; (**C**) Higher-order packaging interactions. Budding of the small (S), medium (M), and large (L) genomic RNA segments, which are likely fully coated with N protein to form RNPs, occurs at the Golgi membrane, and may be promoted by interactions between the RNPs and the RNA dependent RNA polymerase, or L protein (symbolized by black boxes), and the glycoproteins Gc and Gn. The mechanism(s) for selecting one of each of the genomic RNPs per virus particle are still an active area of investigation, and may include RNA-RNA, RNP-RNP, and/or protein-protein interactions.

In the following sections we summarize recent research on genome packaging in the phleboviruses, highlighting the molecular mechanisms that drive the processes. We focus first on the critical binding between a single N protein and its cognate vRNA, then discuss the subsequent RNA packaging that builds upon the nucleocapsid-RNA (N-RNA) interface, including subsequent N protein oligomerization and RNP formation. Finally, the higher order protein and RNA based contributions that mediate RNP packaging into infectious particles are outlined (Figure 2). Understanding the driving force of RNA packaging is critical not only for establishing a complete picture of the molecular virology of the phleboviruses, but also for development of antiviral therapeutics and the exploration of alternate uses of viruses as nanoparticle delivery systems.

## 3. Nucleocapsid-RNA Interactions: RNP Building Blocks

The genomes of negative sense RNA viruses are covered and protected by the viral nucleoprotein or N protein. N proteins fulfill many essential jobs, including protection of vRNA from detection by host defenses and compaction of the genomic RNPs. N protein is required for transcription and replication [29]. N proteins play a role in RNP packaging at the site of assembly through protein-protein interfaces. N proteins are indispensible for the virus. It plays critical roles at several points in the viral replication cycle and is the most abundant viral protein during an infection. It is not surprising that the RNA binding groove of N proteins have been recognized as a potential therapeutic target [37,38,39]. As discussed below, a number of crystal structures of phleboviral N proteins have recently been solved, and have greatly aided our understanding of nucleocapsid protein’s domain structure, interaction with RNA, and the interactions of adjacent N proteins in the ribonucleoprotein structure.

Currently available structural comparisons show very limited similarity amongst nucleocapsid proteins of segmented negative sense RNA viruses classified in the Multinegavirales order, which includes the *Arenaviridae*, *Orthomyxoviridae*, and *Bunyaviridae* families. This is in stark contrast with the capsid proteins of non-segmented viruses belonging to the Mononegavirales, which exhibit much greater homology amongst order members. Even within the *Bunyaviridae*, the structural properties of the N proteins vary widely. For example, the N protein of Crimean Congo hemorrhagic fever virus (CCHFV), of the *Nairovirus* genus, shows greater similarity with the N protein from Lassa virus (LASV), from the *Arenaviridae* family, than it does with RVFV N protein [40]. Although hantavirus nucleoproteins are nearly twice the size of phleboviral N proteins and form trimers in solution, Sin Nombre virus (SNV) N proteins exhibit partial homology with phleboviral N proteins from RVFV, SFTSV, and TOSV [41]. Interestingly, SNV and RVFV nucleocapsid proteins have similar hydrophobic RNA binding pockets [41]. RVFV, TOSV, and SFTSV are the currently available high resolution structural models for phleboviral N proteins, and they indicate that the RNA binding and oligomer formation features are similar amongst phlebovirus members [42,43,44,45,46].

The ~25–30 kDa phleboviral N proteins carry out all of their known essential tasks in the host cytoplasm. Therefore, it has been assumed that N protein must have a mechanism for distinguishing between viral and host RNA binding partners. The two crystal structures available for RVFV may provide clues about how phleboviral N proteins mediate RNA binding. The first published RVFV N protein structure showed a monomeric N protein, devoid of RNA, that did not address the nature of N-RNA binding and N protein oligomerization along the RNA molecule [42]. A second structure where N crystalized in a hexameric architecture in an opened configuration, revealed that N proteins have a helical N-terminal arm that can open and close over the positively charged RNA binding cleft [44]. It became clear that this flexible N-terminal extension was in a closed conformation over the central globular domain in the first crystal structure, perhaps indicating an arrangement that inhibits premature RNA binding following translation of the protein [43,44]. When the N-terminal arm is in an opened state, not only is the RNA binding cleft exposed, but the arm is available to bind a hydrophobic cleft on the outside of a neighboring N protein monomer, mediating oligomerization of N protein monomers into higher order assemblies (Figure 2B). Indeed, earlier biochemical studies showed that N proteins form dimers in solution and that aromatic residues near the amino terminus of the protein are critical for this interaction [47].

Phleboviruses do not encode a phosphoprotein (P), a multifunctional protein in nonsegmented RNA viruses that prevents newly synthesized nucleoprotein from prematurely binding to cellular or vRNA. For example, nascent N proteins of Sendai virus (SeV) and vesicular stomatitis virus (VSV) can form non-specific aggregates with RNA in the absence of bound P. The phosphoprotein helps to maintain N proteins in an unbound state until the nucleoprotein is at the site of virus assembly [48,49,50,51,52,53]. For the phleboviruses, the specificity of the N-RNA interaction and the ability of N protines to oligomerize along the vRNA strand appears instead to be at least partially mediated by accessibility of the RNA binding groove. Although it is currently unclear what triggers the conformational change in N that opens its terminal arm domain and alters its RNA binding state, it is likely that the interaction with RNA or the viral RdRP is important.

Based on the structural data from N-RNA complexes, the energetics of RNA binding to N are dominated by electrostatic and hydrophobic interactions built into the RNA binding groove of N protein. A patch of basic residues that are highly conserved in phleboviruses (R64D, K67D, K74D) flank the central region of the globular N protein body and confirm the location of the RNA binding domain [44]. Mutation of these residues abolishes the ability of N protein to bind RNA [44]. Phlebovirus N proteins sequester bound RNA bases within a deep positively charged groove with the sugar phosphate backbone engaging with the positively charged residues, effectively rendering the RNA inaccessible to RNase degradation [43]. For TOSV and RVFV, the crystal structures predict little specificity in the RNA binding cleft and reveal that the N-RNA interaction can be indiscriminate, as N proteins crystalize in higher order structures with long stretches of generic polyU RNA [43,46]. However, it is suspected that N proteins engage in both specific and non-specific RNA binding modes, since many studies have demonstrated that bunyaviral N proteins preferentially recognize and bind vRNA sequences [54,55,56,57]. Furthermore, in vitro selection of RNA aptamers bound by RVFV N protein reveals highly redundant sequences and structural motifs that exhibit similarity with several regions along the viral genome [58]. It is likely that N protein engages in an initial specific binding event when N proteins are stimulated to open its N-terminal arm, but that subsequent binding is mediated by the N-terminal arm contacts with neighboring N proteins. Thus, N proteins oligomerize on vRNA via non-specific, electrostatically driven RNA binding guided by protein-protein interactions of N protein monomers.

## 4. RNP Formation and Trafficking

The RNP is the vehicle that protects, transports, and packages the viral genomic RNA. RNPs serve as the templates for the synthesis of mRNA, cRNA, and new copies of the vRNA. Phleboviral RNPs are built from individual N-RNA interactions and form circularized pseudo-helical structures with the 5′ and 3′ complementary ends of the S, M, or L segments interacting via base pairing and bound by the L protein. It has yet to be determined how small planar crystalized RNPs (e.g., the RVFV hexamer discussed earlier) are expanded to generate full-length native RNP complexes that coat each genome segment. Recent work on TOSV indicates that RNA binding stimulates conformational changes at the protein interface between adjacent N proteins that causes rotation between N subunits [46]. This rotation allows the formation of a continuous RNA binding tunnel as longer RNAs are encapsidated by N. The flexible N-terminal arm of the nucleocapsid protein is apparently a shared feature with several other segmented negative sense RNA viruses, and in addition to impacting N-RNA binding specificity and oligomerization properties, the N-terminal arm facilitates an RNP architecture that is very elastic. This flexibility allows RNPs to adopt a variety of geometries that are likely essential for RNP formation and shape, compaction and packaging into progeny virions [59].

The direct mechanism by which the RNPs are guided to the site of viral budding at the Golgi compartment remains unclear. Bunyaviral RNPs have been shown to accumulate in cytoplasmic aggregates for hantaviruses, tospoviruses and nairoviruses, implying that the RNPs alone do not localize to the Golgi [60,61,62]. Rather, the force driving the RNPs to the site of assembly appears to be based on protein-protein interactions between the N protein and the viral glycoproteins, as discussed below.

In many viruses, viral matrix proteins serve as the master mediators of virion assembly and provide structural integrity for budding virus particles. Matrix proteins function by bridging the interaction between the genomic RNPs with the glycoproteins in the lipid envelope and may intrinsically induce membrane curvature or even hijack host protein budding scaffolds. Bunyaviruses are unique amongst other negative-sense RNA viruses in that they do not encode a matrix protein, suggesting an essential role for the viral glycoproteins in concert with the nucleocapsid proteins in anchoring the RNPs into the budding virion framework [63].

The viral envelope glycoproteins Gn and Gc are cotranslationally cleaved from a polyprotein precursor to form the mature proteins encoded within the M segment [64,65,66]. When expressed individually, only Gn is transported to the Golgi, while Gc is retained in the endoplasmic reticulum [67]. In the ER, Gc must physically interact with and heterodimerize with Gn, which contains the Golgi localization signal, in order to be transported and retained at the site of virion production [67]. As type I integral membrane proteins, the glycoprotein C-terminal tails extend towards the cytoplasmic side of the membrane, while the N-terminal ectodomains form spikes that stud the virion outer envelope. The *Bunyaviridae* possess large Gn C-terminal tails that extend outward on the cytoplasmic face of the Golgi membrane. Enzymatic digestion of membrane bound Karimabad virus (KARV) glycoproteins during infection indicated that around 12% of the glycoproteins were readily digested, suggesting that a large portion of the Gn-Gc complex is not membrane bound and is thus accessible for engaging in protein-protein interactions with the N proteins [68].

Given the lack of a matrix protein within this virus family, it is not surprising that many lines of evidence point to the association of N protein with Gn and Gc. For example, in UUKV infected cells, Gn/Gc can be co-immunoprecipitated with the N protein [69]. Tunicamycin treatment, which inhibits N-linked glycosylation and stalls Gn and Gc in the rough ER, illustrates that this interaction occurs early, because nucleocapsid protein also remains associated with the glycoproteins under these conditions [69]. Electron microscopy of cells infected with Punta Toro and Karimabad viruses shows that RNPs coalesce at Golgi regions studded with glycoprotein spikes [68]. Single particle cryo-electron microscopy (cryo-EM) of RVFV shows RNP densities just below the membrane, also indicating an interaction between N and the glycoprotein tails [70].

Efficient RNP packaging in RVFV and UUKV minigenome and virus like particle (VLP) systems requires a direct interaction between the Gn cytoplasmic tail and N protein [71,72]. Mutagenesis of the UUKV virus C-terminal Gn tail confirms the critical residues that interact with UUKV N [71]. Gc is important for Gn expression and efficient virus production, although no direct role in RNP packaging has been established [72]. However, pleiomorphic VLPs are produced from baculovirus vector infected insect cells expressing only Gc and N, indicating that there may also be an interaction between Gc and N [73].

Evidence indicates that Gn is also responsible for recruiting the viral polymerase to the site of assembly at the Golgi. The bunyaviral RdRP is synthesized from the L segment on cytosolic ribosomes and maintains a diffuse cytoplasmic localization in the absence of other viral proteins. In RVFV, when Gn is expressed with the RdRP, the polymerase colocalizes with Gn [72]. Conversely, Gc expression with the RdRP has no effect on the RdRP localization [72]. Furthermore, the region required for RdRP recruitment activity was traced to a terminal domain in the Gn cytoplasmic tail [72]. Surprisingly, of the proteins that comprise RVFV virions, experimental evidence indicates that only the RdRP is dispensable for efficient VLP production, although RdRP packaging is absolutely required for producing progeny virions capable of synthesizing primary transcripts in newly infected cells [72].

Interestingly, phleboviruses are the only genus within the *Bunyaviridae* whose Gn proteins lack zinc-finger (ZF) domains in their cytoplasmic tails. CCHFV and Andes virus (ANDV) Gn proteins each have dual ZF with the traditional ββα fold, but they are positioned close together so that they form a globular domain, which may be important for RNA recognition and/or for RNP binding [74,75,76]. Interestingly, VLP formation occurs in the presence of the glycoproteins alone for both UUKV and RVFV, implying that RNPs are not prerequisite for particle budding and that local glycoprotein concentration can mediate the budding process [72,77]. This phenomenon of particle formation in the absence of RNPs has not been confirmed outside of the phleboviruses. However, it remains to be determined whether any members of the phleboviruses, or any other members of *Bunyaviridae*, recruit host factors that play a role in the viral budding process.

## 5. RNP Packaging: Higher Order Assembly

Viral genome assembly strategies are strikingly diverse but can be inferred based on the characteristics of the nucleic acid to be packaged. For example, the steric and charge constraints conferred by viral dsDNA (e.g., bacteriophage, poxviruses, adenoviruses, herpesviruses) genomes results in their packaging into preformed protein capsid shells using viral molecular motors driven by ATP hydrolysis. In contrast, more flexible single stranded RNA genomes can be manipulated into diverse conformations driven by protein-RNA interactions. The non-enveloped positive sense single stranded tobacco mosaic virus (TMV) RNA genome can assemble into rigid rod-like helical capsids in vitro via spontaneous electrostatic interactions between the capsid protein and vRNA [78]. Likewise, many enveloped negative sense ssRNA viruses, such as VSV and respiratory syncytial virus (RSV), assemble their nucleoproteins sequentially around their genomes to form helical or pleiomorphic capsids, which are subsequently condensed and packaged into highly uniform bullet shaped virions at the plasma membrane. This morphology was thought to be driven by ordered interactions with viral matrix proteins, but recent cryo-EM evidence suggests the RNA-N complex itself can self-assemble into these bullet shaped structures [79].

Genome packaging increases in complexity for members of the *Bunyaviridae*, *Orthomyxoviridae* and *Arenaviridae* families, which are also ssRNA viruses, but encode multiple genome segments. In order for a segmented RNA virus to be infectious, a nascent virus particle must contain at least one copy of each genome segment. This implies that RNP packaging is not only contingent upon differentiation between viral and host nucleic acids, but also between diverse viral nucleic acids, including sense and antisense genome. While genome segmentation offers evolutionary advantages, it clearly creates the need for increased specificity in genomic packaging to ensure production of viable virions. The possibility of acquiring beneficial traits via reassortment must therefore outweigh this cost of increasing complexity conferred by genome segmentation.

Historically, arguments have been posed in support of both segment-specific and random packaging mechanisms for multi-segmented RNA viruses. Eight negative sense single stranded RNA genome segments must be incorporated into infectious progeny of the prototypical orthomyxovirus, influenza A (IAV). When compared with VSV mentioned above, IAV also forms helical structures, but there is a non-uniform association of RNPs with the matrix proteins, resulting in a diverse pool of viral particles. However, observed IAV particle:PFU ratios are significantly lower than statistical estimates based on random chance genome incorporation models, indicating that there must be some degree of specificity inherent in the gathering and parceling of all eight segments. Likewise, the tri‑segmented Bunyamwera virus, of the *Orthobunyavirus* genus, exhibits a particle:PFU ratio of 2.6–7.2, further implying that viruses have built in mechanisms to ensure their genomes are packaged into nascent particles [80].

Within the phleboviruses, our understanding of RNP co-packaging is only beginning to take shape. In addition to the overall packaging mechanism (random versus segment specific), an active area of research is focused on whether the characteristics of one segment make that segment more likely to be incorporated into progeny virus particles over another. There are abundant examples illustrating the existence of RNA based packaging signals across many diverse families of viruses that impact genome packaging. Even in the model bacteriophage MS2 system, positive sense RNA genome assembly is driven by coat protein recognition of degenerate RNA packaging signals [81]. RNA signals can exist in coding and non-coding regions, and consist of primary sequences and/or secondary and tertiary structures. Their functional activity may occur via interaction with host or viral proteins or through intra- or inter-segment specific RNA-RNA interactions.

Recent studies in IAV indicate that for each segment, RNA packaging signals interact with other vRNA elements in coding and noncoding regions to facilitate segment specific co-incorporation [82,83,84,85,86,87,88,89]. Influenza virions, however, exhibit RNP densities indicative of organized RNP densities with seven RNPs surrounding a central eighth RNP [90,91]. In contrast, cryo-EM of model phleboviruses UUKV and RVFV give no such indication of distinctly separate RNP densities, but show highly ordered interactions between N and the glycoprotein tails [92]. As of yet, no direct evidence has been shown for RNA based inter-segment packaging interactions. Given that the BUNV particle to PFU ratio approaches one [80], each particle likely contains genomic RNA, although perhaps not one of each L, M, and S segment. Ratios of genome segments are somewhat consistent within virions of model genus members (RVFV L:M:S = 1:3.9:3.9; UUKV L:M:S = 1:4:2), yet these ratios suggest S and M are more prevalent than L in the total population of virions collected from supernatants [24,93]. It seems improbable that individual particles routinely contain such a skewed ratio of S and M to L segments but it remains a technical challenge to catalog the RNAs contained within single virus particles.

It has also been shown that S antigenomic RNAs are packaged in UUKV virions and all three antigenomic segments may be packaged in RVFV particles [21,94]. Work using a RVFV reverse genetics system to alter the coding strategy in the ambisense S segment suggested the possible presence of a *cis*-acting signal within the coding region that determines whether sense or antisense genome is packaged. In this study the 5’ and 3’ non-coding regions (NCRs) were left intact, but the NSs and N protein open reading frames were swapped. In the RVFV MP12 virus more genomic S RNA was packaged, however in the MP12 clone with the coding regions reversed, more antigenomic S was packaged [95].

Experimental results also show that there is genome packaging variation across species and cell lines. For example, multiple viable four-segmented RVFV variants have proven capable of spreading, albeit with reduced growth and virulence, in mammalian cell lines, but are unable to propagate in mosquito cells [96]. In the RVFV variant in which N is expressed from the NSs locus and NSs from the N locus, growth is attenuated in mammalian cells but resulted in cell death in mosquito cells [95]. These results raise intriguing questions and indicate that host factors may impact viral genome packaging.

## 6. RNA Based Packaging Signals in NCRs

Bunyaviral genome NCRs have genus specific complementary sequences that can base pair to form the panhandle shaped RNPs and contain essential replication promoter elements, transcription and encapsidation signals. Given these already established essential roles, the NCRs are a logical place to search for packaging signals. In fact, Murakami and coworkers demonstrated a strong overlap between the packaging and replication signals within the terminal 15–25 nts comprising the panhandles of the S, M, and L segments in RVFV [97]. Minireplicons are useful tools for investigating the influence of the NCRs on segment specific packaging. These systems utilize intact NCRs flanking reporter genes so that delivery of genetic material can be monitored in subsequent passages. Several studies using bunyaviral minireplicons indicate that intact NCRs are necessary and sufficient for packaging minireplicon RNAs into virions [98,99,100]. Furthermore, there are critical packaging elements that exist in the NCRs and segment-specific packaging competencies vary, as addressed below.

L segment based minigenomes exhibit the strongest packaging efficiencies compared to the S and M segments for both UUKV and BUNV viruses. Reporter activity following serial passage of virus minireplicon genomic RNAs packaged into virions shows that L is maintained for several passages and M is retained for fewer passages, while the S signal rapidly decreases [99,101]. Interestingly, inclusion of the coding region of the S segment did not improve S packaging efficiency [101]. This implies, at least for UUKV and BUNV, that the L segment may have stronger packaging signals located within its NCRs. Although BUNV minigenome NCRs exhibit differential promoter activities (M > L > S) [102], a BUNV recombinant virus with M segment NCRs flanking the L segment coding region produced attenuated virus with less L expression [80]. This not only verifies that packaging is not just a direct result of genome quantity derived from differential promoter activity in minireplicons, but more importantly, it indicates that the NCRs are not the only variable playing a role in segment co-packaging.

Studies using RVFV suggest that in infected cells, vRNA accumulates S > M > L [24]. It has been suggested for RVFV that the M segment coordinates copackaging of the S and L segments [103]. M and M NCR deletion minigenome segments were packaged into VLPs with similar efficiencies but only the M segment with an intact NCR could co-package the S segment. While L packaging was not dependent on the M segment, L was only efficiently packaged into VLPs in the presence of both S and M [103]. However, subsequent studies have demonstrated that the RVFV M segment is not required for packaging of L and S into RVFV particles [104,105,106]. Furthermore, a bi-segmented RVFV that includes the glycoprotein genes in the S segment, in place of the non-essential NSs, and lacks an M segment altogether was shown to be viable [107]. Conflicting results, however, may indicate that the presence of coding regions might be critical, if not for maintaining the putative RNA signals within these regions, then perhaps for keeping the length of each segment consistent with the actual genome. Viruses are under selective pressure to maintain an optimal genome length. Genome length is the result of a delicate balance between genetic economy and RNP stability and charge distribution. Maintenance of genome length is less frequently considered in minireplicon experimental design, but perhaps deserves attention.

## 7. Conclusions

The work summarized above indicates that there is both innate flexibility and variability in the RNA packaging mechanisms employed by phleboviruses. The initial and most fundamental event in RNA packaging is the interaction between the viral nucleocapsid protein and its cognate RNA, since subsequent interactions build upon this base. Within the past few years there has been a wealth of structural and biochemical data that show the N-RNA interaction can be both specific and promiscuous. Future work should focus on identifying the molecular triggers that control the RNA binding modes of N protein. We know surprisingly little about the nature of phleboviral RNPs. We do not know if co-packaging of RNPs is driven by protein-protein interactions or via nucleic acid interfaces. The NCRs are critical for packaging, but RNA genomic elements that mediate segment co‑packaging have yet to be identified. In light of recent research demonstrating the importance of inter-segment signaling in segmented RNA viruses, we can be certain that there are phleboviral co‑packaging signals awaiting discovery. Segmented RNA viruses must be rapidly adaptable to new environmental challenges and stimuli, and also not so rigid in their packaging strategies that they ablate the possibility of creating genetic reassortants. Thus, it is hardly surprising that there is a fundamental plasticity in the RNA packaging mechanisms exploited by these viruses.
